# Thyroid function, physical activity and sedentary behaviour: A bidirectional two-sample Mendelian randomisation study

**DOI:** 10.7189/jogh.14.04154

**Published:** 2024-09-27

**Authors:** Chenyu Zhang, Yutong Han, Xiaotong Gao, Weiping Teng, Zhongyan Shan

**Affiliations:** 1 Department of Endocrinology and Metabolism, Institute of Endocrinology, NHC Key Laboratory of Diagnosis and Treatment of Thyroid Diseases, The First Hospital of China Medical University, Shenyang, Liaoning, China; 2 The First Affiliated Hospital of China Medical University, General Medicine, Shenyang, Liaoning, China

## Abstract

**Background:**

The interinfluence of thyroid function and daily physical activity (PA) remains unclear. We examined the causal relationship between genetically proxied thyroid-related traits; hypothyroidism, hyperthyroidism, thyroid stimulating hormone (TSH) and free thyroxine (FT4), and daily PA measures; leisure screen time (LST) and moderate-to-vigorous physical activity (MVPA), using Mendelian randomisation (MR) analysis.

**Methods:**

We used genome-wide association study (GWAS) data from the ThyroidOmics Consortium and the most comprehensive meta-analysis on PA, comprising data on hypothyroidism (n = 53 423), hyperthyroidism (n = 51 823), TSH within the reference range (n = 54 288), fT4 within the reference range (n = 49 269), LST (n = 526 725), and MVPA (n = 608 595) to conduct a bidirectional two-sample MR analysis. The inverse variance weighted (IVW) method was employed as the primary result. Sensitivity analyses included MR-Egger, weighted median, and MR pleiotropy residual sum and outlier (MR-PRESSO) regression. Similar investigations were conducted in the reverse direction. Finally, we analysed a multivariable MR using body mass index (BMI)-related traits GWAS data.

**Results:**

In the primary IVW analysis, an increase in genetically proxied TSH levels significantly increased LST (correlation coefficient (*β*) = 0.040; 95% confidence interval (CI) = 0.020–0.061, *P* = 9.776 × 10^−5^). The multivariable MR analysis indicated that the positive causal effect still existed when considering the influence of BMI (MVMR-IVW: β = 0.042; 95% CI = 0.011–0.073, *P* = 0.007). Conversely, there was no evidence to suggest that PA impacts thyroid function.

**Conclusions:**

The results of this MR analysis suggest that thyroid function influences daily PA. The positive association between TSH and LST is not confounded or mediated by BMI.

Thyroid disease is one of the most common endocrine disorders and may result in thyroid dysfunction (hypothyroidism and hyperthyroidism), severely affecting the patient's quality of life. Insufficient physical activity (PA) is a prevalent health problem in modern society that increases the risk of cardiovascular diseases and cancer [1] and shortens life expectancy [2]. Sedentary behaviour (SB) is defined as any waking behaviour characterised by an energy expenditure of 1.5 metabolic equivalents or less, and leisure screen time (LST) is the most widely studied form of SB [1,3]. Reducing SB and increasing moderate-to-vigorous intensity physical activity (MVPA) during leisure time can significantly improve health [4].

Thyroid function is closely related to PA capacity. Thyroid hormones (THs) are important regulators of energy metabolism that can affect PA through multiple systems. THs can affect skeletal muscle activity through the Na/K-ATPase enzyme, improving strength, balance, and range of motion [5]. THs can increase cardiac contractility, cardiac output, and work [6], and can promote erythropoietin elevation, which helps with oxygen supply to blood tissues [7]. When there is an excess of THs, such as when athletes supplement it, their physical performance significantly improves [8]. If the excess is severe, it may manifest as periodic paralysis [9]. When TH deficiency leads to hypothyroidism, patients may show severe muscle weakness, limiting the capacity for intense activity [10]. Some studies suggest that, after TH replacement therapy, heart function and cardiac output in hypothyroidism patients can be improved [11].

However, PA capacity does not always correspond to daily PA level. In fact, research on the relationship between thyroid function and PA/SB has produced inconsistent results. For example, recent results of a large cohort study showed that endogenous TH levels were not associated with overall PA [12]. A cross-sectional study showed that female patients receiving levothyroxine treatment still had insufficient PA [13]. However, previous studies have shown that, in the US National Health and Nutrition Examination Survey (NHANES) data set, active adult T4 levels were lower, while the slope of TSH/T4 was decreased [14]. A case-control study using PA monitors to evaluate the physical activity levels of healthy female patients with subclinical hypothyroidism found that those with subclinical hypothyroidism had lower PA levels [15]. A survey of healthy elderly men found that older people with SB had higher TSH levels [16]. These observational studies have produced inconsistent results, and whether there is a causal relationship between thyroid function and PA/SB still requires further exploration. Moreover, available studies suggest that SB can increase the risk of some endocrine system diseases, such as type 2 diabetes [17] and metabolic syndrome [18]. Obesity is known to affect the hypothalamic pituitary thyroid axis through leptin [19]. Whether PA in turn affects thyroid function is not known.

Mendelian randomisation (MR) analysis follows the Mendelian law of inheritance of random assignment of parental alleles to offspring by using instrumental variables (IVs) as a tool to represent exposure; in doing so, it aims to assess the causal relationship between exposure and outcome risk [20]. In this study, we hypothesised that there is a potential causal relationship between thyroid function and PA/SB. We assessed the bidirectional causal relationship between thyroid disease traits (hyperthyroidism and hypothyroidism), thyroid hormone level traits (TSH and FT4), and two PA/SB-related indicators (LST and MVPA) using two-sample MR tests with the most comprehensive thyroid disease consortium data [21] and the most recent large-scale genome-wide association study (GWAS) meta-analysis data for PA/SB [22]. We also further explored the potential role of body mass index (BMI) in these associations.

## METHODS

### Study design

**Figure 1:**
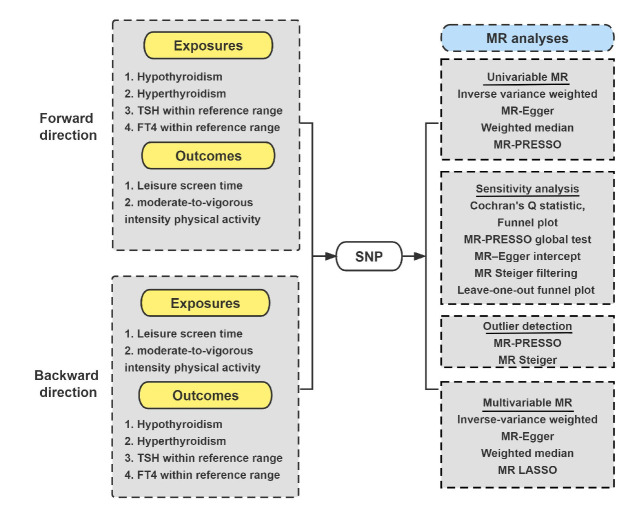
Study design and hypothesis overview. FT4 – free thyroxine, MR – Mendelian randomisation, MR-PRESSO – Mendelian randomisation pleiotropy residual sum and outlier; SNP – single nucleotide polymorphism, TSH – thyrotropin.

We followed the STROBE-MR guidelines [20] in reporting our findings below (Table S1 in the **Online Supplementary Document**). The IV analysis mimics a randomised controlled trial by randomly assigning single nucleotide polymorphisms (SNPs) (removing the effect of confounding factors) [23]. Furthermore, such an MR study must satisfy three assumptions for each IV; it must predict the exposure of interest; be independent of potential confounders; and influence the outcome only through risk factors [24]. In the forward direction, we first extracted genetic variants of thyroid-related traits as IVs. Second, we collected the full SNP summary data from GWAS associated with LST and MVPA. Third, we performed four univariable MR analyses and a series of sensitivity analyses. Fourth, we performed multivariable MR analysis to investigate the role of BMI in the relationship between thyroid function and PA/SB. We also conducted a similar analysis in the reverse direction (**Figure 1**).

### Data collection

We drew the data for hyperthyroidism, hypothyroidism, TSH, and FT4 from The ThyroidOmics Consortium [21] (**Table 1**). People of non-European ancestry, those who used thyroid medication (defined as Anatomical Therapeutic Chemistry-ATC code H03), or had previous thyroid surgery were excluded. To obtain a comprehensive and reliable conclusion, we obtained the LST and MVPA data from the most recent GWAS meta-analysis on the GWAS catalog website [22,29]. We then adjusted the data for age, age squared, principal components reflecting population structure, and additional study-specific covariates.

**Table 1:** Details of the data sources included in the MR analysis*

Phenotype	Description	Study type	Participants	Source
Self-reported physical activity and sedentary traits				
*LST*	Time spent watching TV, playing videogames, sitting at the computer, etc.	Meta-analysis of genome-wide association analyses	526 725 (discovery stage); up to 703 901 subjects in meta-analysis	GWAS study GCST90104339 [25]
*MVPA*	Moderate-to-vigorous intensity physical activity during leisure time, such as swimming, jogging, etc.	Meta-analysis of genome-wide association analyses	608 595 (discovery stage); up to 703 901 subjects in meta-analysis	GWAS study GCST90104341 [26]
Thyroid dysfunction				
*Hyperthyroidism*	TSH level lower than the reference range. Individuals who were receiving thyroid medication or had a history of thyroid surgery were excluded.	Case-control GWAS meta-analysis	Total: 51 823, cases: 1840, controls: 49 983	ThyroidOmics Consortium, GWAS meta-analysis summary statistics [27]
*Hypothyroidism*	TSH level higher than the reference range. Individuals who were receiving thyroid medication or had a history of thyroid surgery were excluded.	Case-control GWAS meta-analysis	Total: 53 423, cases: 3440. controls: 49 983	ThyroidOmics Consortium, GWAS meta-analysis summary statistics [27]
Thyroid hormone level traits				
*TSH*	TSH level within the cohort-specific reference range.	Meta-analysis of the ThyroidOmics consortium	54 288 (discovery stage); Up to 72 167 subjects in meta-analysis	ThyroidOmics Consortium, GWAS meta-analysis summary statistics [27]
*FT4*	FT4 level within the cohort-specific reference range.	Meta-analysis of the ThyroidOmics consortium	49 269 (discovery stage); up to 72 167 subjects in meta-analysis	ThyroidOmics Consortium, GWAS meta-analysis summary statistics [27]
Multivariate MR analysis				
*BMI*		Meta-analysis of the UK Biobank and GIANT Consortium Summary Statistics	807 000 subjects in meta-analysis	GIANT Consortium, data file [28]

BMI – body mass index, FT4 – free thyroxine, GIANT – Genetic Investigation of ANthropometric Anthropometric Traits, GWAS – genome-wide association study, LST – leisure screen time, MR – Mendelian randomisation, MVPA – moderate-to-vigorous intensity physical activity during leisure time, TSH – thyroid stimulating hormone

*All data sources included in the analysis have 100% European ancestry.

A previous study suggests that BMI could be a confounding factor affecting thyroid function and PA [30]. We therefore also retrieved BMI GWAS data for multivariable MR analysis from the UK Biobank and GIANT Consortium [28,31] (**Table 1**). To reduce heterogeneity, we selected all individuals from European populations. We found no significant overlap between the exposure group data set and the outcome group data set.

### IV selection criteria

**Figure 2:**
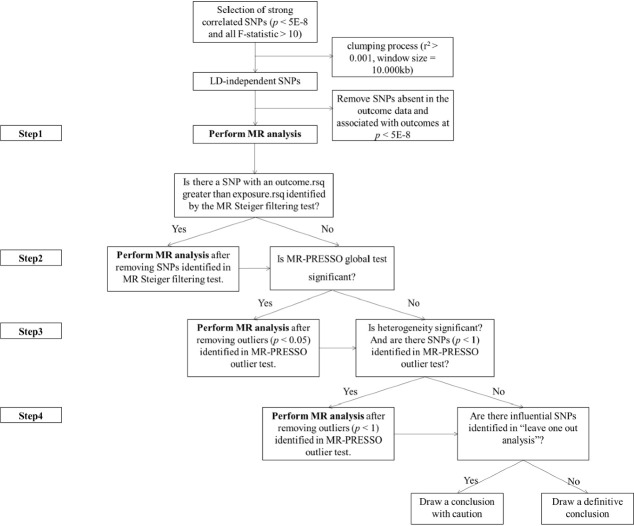
Flowchart of the selection process for instrumental variables and step-by-step execution analysis. Step 1: MR analysis with the complete selected SNPs. Step 2: MR analysis after removing the SNPs with FALSE in the MR Steiger test of directionality. Step 3: MR analysis after removing the SNPs with *P*-value less than threshold (0.05) in the MR-PRESSO outlier test. Step 4: MR analysis after removing all the SNPs with *P*-value less than 1 in the MR-PRESSO outlier test. MR – Mendelian randomisation, MR-PRESSO – Mendelian randomisation pleiotropy residual sum and outlier, SNP – single nucleotide polymorphism.

We identified the IVs by the following criteria: SNPs at the genome-wide significance level (*P* < 5 × 10^−8^), SNP clumping using the PLINK algorithm (r^2^ = 0.001 and window size = 10 000 kb), and removal of SNPs absent in the outcome data and associated with outcomes (*P* < 5 × 10^−8^) (**Figure 2**). We also calculated F-statistics were calculated using the formula F = (β/standard error (SE))^2^ [32] and *R*^2^ using the formula: *R*^2^ = 2 × (1 − minor allele frequency (MAF)) × MAF × β^2^ or *R^2^* = 2 × (1 − MAF) × MAF × (β/standard deviation (SD))^2^ [32]. The F-statistics of all SNPs were greater than 10 (Table S4 and S8 in the **Online Supplementary Document**).

### MR analysis

We used four MR analysis methods to assess the causal effects between thyroid function and PA/SB. Our main analytical approach was to use the inverse variance weighted (IVW) method. The IVW regression analysis first entailed calculating a Wald estimate for each SNP (i.e. the β of the exposure SNP divided by the β coefficient of the outcome SNP). We also used a combination of meta-analysis methods to obtain an overall estimate of the effect of exposure on the outcome [32]. The IVW does not account for the presence of the intercept term and is fitted with the inverse of the ending variance (standard error quadratic) as the weight. If horizontal pleiotropy is not present or is balanced, a reliable causal estimate can be obtained by IVW analysis [33]. When there was heterogeneity, we used the IVW random effects model. The weighted median method can provide consistent estimates when more than 50% of the weight comes from valid instrument variants. Compared to the MR pleiotropy residual sum and outlier (MR-PRESSO) test, the weighted median method has less bias in the causal estimation, but also less precision [34]. MR-Egger regression is assumed to be based on instrumental strength independent of direct effect, which ensures a valid test of the null causal hypothesis and could provide a test for unbalanced pleiotropy and considerable heterogeneity. However, it requires a larger sample size for the same analysis and may be strongly influenced by outlier genetic variants resulting in inaccurate results. When there is pleiotropy, the results calculated by the MR-Egger method are preferred [35]. MR-PRESSO consists of three parts; a detection of horizontal pleiotropy (global test), correction for horizontal pleiotropy by outlier removal (outlier test), and testing for significant differences in causal estimates before and after outlier correction (distortion test) [34]. It is a method for identifying and correcting outliers in IVW regression. The outlier test requires that at least 50% of the IVs be valid, that there is balanced pleiotropy, and that the instrumental strength independent of the direct effect requirement (i.e. that instrument exposure and pleiotropy effects are irrelevant) is met. When the percentage of horizontal pleiotropy is small (≤10%), the MR-PRESSO outlier-adjusted causal estimates have better precision than MR-Egger. However, the opposite is true when the percentage of horizontal pleiotropy is high (≥50%) [34]. MR-PRESSO may be sensitive to outliers and noise in the input data; although it aims to detect and correct this, it may not eliminate their impact in practice [34]. The method is also complex and computationally intensive, especially when dealing with large-scale genetic data [34]. This may limit its application in large-scale studies. The combined use of these methods makes the test results more robust and reliable.

The sensitivity analysis included Cochran's Q statistic, the MR-Egger intercept test, the MR Steiger filtering method, the MR-PRESSO global test, as well as scatter, funnel, and leave-one-out plots (**Figure 2**). We used Cochran's Q statistic to determine heterogeneity, where a *P*-value <0.05 was regarded as significant heterogeneity. We used the MR-Egger intercept to assess and examine the presence of directional pleiotropy [35]. We identified and excluded SNPs that may lead to reverse causal relationships using the MR Steiger test of directionality and conducted repeated MR analyses [36]. Next, we used the MR-PRESSO global test to identify and remove SNPs with pleiotropic effects [34]. We repeated the MR analysis after excluding the outliers if there were pleiotropic outliers. At this point, if heterogeneity still existed, we chose to perform MR analysis under the condition of removing all SNPs with a *P*-value <1 in the MR-PRESSO outlier test [37]. We also used leave-one-out analyses to exclude SNPs one by one to determine the robustness of the results; scatter plots to visually observe the directionality of the different MR analysis methods; and funnel plots to assess the presence of discrepant SNPs. The detection of an SNP with potential impact through the leave-one-out approach meant that caution should be exercised in drawing conclusions.

### Statistical analysis

We adopted a Bonferroni-corrected threshold of *P* < 0.00625 (α = 0.05/8) to interpret the multiple tests in our main MR analysis. The MR estimation results are presented as correlation coefficients (β) or odds ratios (ORs) with their corresponding 95% confidence intervals (CIs). We conducted data analysis using R, version 4.3.1 (R Core Team, Vienna, Austria) and applied the R packages ‘TwoSampleMR’ [36], version 0.5.6, ‘MendelianRandomization’ [38], version 0.8.0, and ‘MRPRESSO’ [34], version 1.0.

### Ethical approval

All relevant GWAS data had already been approved by the respective institutional ethics review boards; therefore, no additional ethical review was required for the published data of this MR study.

## RESULTS

### MR analysis of thyroid-related traits on PA/SB phenotypes (forward direction)

According to the IV selection criteria, we screened 12, 15, 45, and 24 SNPs at *P* < 5 × 10^−8^ and *r*^2^<0.001 for hypothyroidism, hyperthyroidism, TSH and FT4 (Tables S3A–D in the **Online Supplementary Document**), respectively. If, during the extraction of specific SNPs from the outcome GWAS, the target SNP was not present in the outcome and could not be searched from the outcome GWAS with the requested SNP as a proxy in the LD, the SNPs were to be excluded.

**Figure 3:**
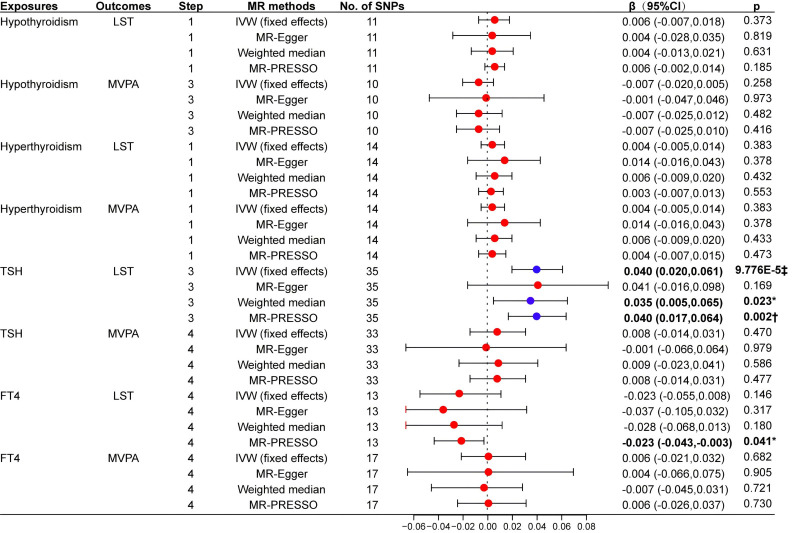
Univariate MR analysis evaluating the causal effects of thyroid-related traits on LST and MVPA. Step 1: MR analysis with the complete selected SNPs. Step 2: MR analysis after removing the SNPs with *P value*<0.05 in the MR-PRESSO outlier test. Step 4: MR analysis after removing all the SNPs with *P*-value <1 in the MR-PRESSO outlier test. FT4 – free thyroxine, LST – leisure screen time, IVW – Inverse variance weighted, MR – Mendelian randomisation, MR-PRESSO – Mendelian randomisation pleiotropy residual sum and outlier, MVPA – moderate-to-vigorous intensity physical activity during leisure time, SNPs – single nucleotide polymorphisms, TSH – thyrotropin. *P < 0.05. †*P* < 0.01. ‡*P* < 0.001.

**Figure 4:**
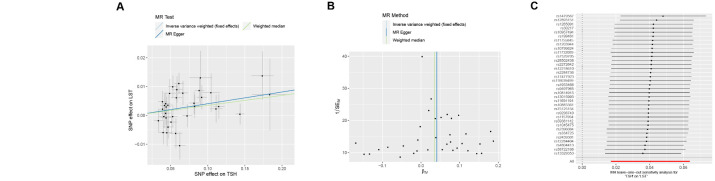
Scatter plots, funnel plots, and plots of leave-one-out analysis for MR analysis of the causal effect between TSH and LST. **Panel A.** Scatter plots for MR analysis of the causal effect between TSH and LST. **Panel B.** Funnel plots for MR analysis of the causal effect between TSH and LST. **Panel C.** Plots of leave-one-out analysis for MR analysis of the causal effect between TSH and LST. LST – leisure screen time, MR – Mendelian randomisation, TSH – thyrotropin.

The IVW analysis indicated that an increase of one SD in genetically proxied TSH (within the normal range) was significantly associated with increased LST (β = 0.040; 95% CI = 0.020, 0.061, *P* = 9.776 × 10^−5^) (**Figure 3**, Table S4 in the **Online Supplementary Document**). Similarly, the MR-PRESSO analysis also indicated a significant association (β = 0.040; 95% CI = 0.017–0.064, *P* = 0.002) (**Figure 3**), whereas the weighted median method returned nominally significant results (β = 0.035; 95% CI = 0.005, 0.065, *P* = 0.023). There was no significant directional pleiotropy according to the MR–Egger regression (*P >* 0.987) (Table S2 in the **Online Supplementary Document**). The scatter plot showed that all MR analysis methods had the same direction of effect; the funnel plot was symmetrical, confirming that all outliers were removed; while the leave-one-out plot indicated that the causal relationship between TSH and LST was robust and not influenced by a single SNP (**Figure 4**, panels A−C). The MR Steiger method results showed no reverse causality for the included SNPs (Table S4 in the **Online Supplementary Document**).

Moreover, genetically proxied hypothyroidism, hyperthyroidism, and serum FT4 levels were not causally related to either LST or MVPA in any of the IVW analyses. However, the MR-PRESSO regression analysis showed a nominally significant negative association between genetically determined increased FT4 levels and LST (β = −0.023; 95% CI = −0.043–, −0.003, *P* = 0.041) (**Figure 3**; Table S5 in the **Online Supplementary Document**). Similarly, we did not find a causal relationship between genetically proxied serum TSH levels and MVPA when using any of the methods. In the sensitivity analysis, neither the MR-Egger intercept nor the MR-PRESSO global test was statistically significant (Table S2 and S5 in the **Online Supplementary Document**).

**Figure 5:**
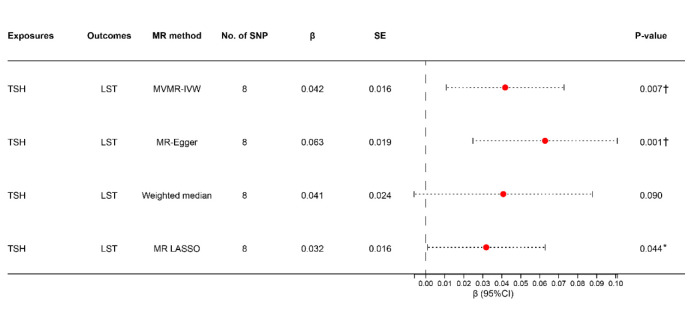
Univariate and multivariable MR analyses evaluating the causal effects of TSH and BMI on LST. BMI – body mass index, LST – leisure screen time, IVW – Inverse variance weighted, MR – Mendelian randomisation, MR-PRESSO – Mendelian randomisation pleiotropy residual sum and outlier, TSH – thyrotropin. *P < 0.05. †*P* < 0.01. ‡*P* < 0.001.

We subsequently performed MVMR to investigate the role of BMI in the relationship between thyroid-related traits and PA/SB. The data related to the SNPs included in the analysis are presented in Table S6 in the **Online Supplementary Document**. We found that the association between genetically proxied TSH and the risk of LST observed in the univariable MR analysis remained consistent in the MVMR analysis (**Figure 5**). Genetically proxied increases in TSH had a significant positive causal relationship with LST in three MVMR methods: MVMR-IVW (β = 0.042; 95% CI = 0.011, 0.073, *P* = 0.007), MR-Egger (β = 0.063; 95% CI = 0.025, 0.101, *P* = 0.001), and MR-Lasso (β = 0.032; 95% CI = 0.001, 0.063, *P* = 0.044) (**Figure 5**). However, the effects on LST risk were not significant when accounting for the effect of BMI in the weighted median method (β = 0.041; 95% CI = −0.006, 0.088, *P* = 0.090) (**Figure 5**). Since we used the IVW analysis results as the primary basis for our MR analysis, we concluded that there was a relationship between TSH and LST in the MVMR analysis. Cochran's Q statistic and MR-Egger intercept results showed no significant directional pleiotropy and heterogeneity (Table S7 in the **Online Supplementary Document**).

### MR analysis of PA/SB phenotypes on thyroid-related traits (reverse direction)

Under the screening conditions of *P* < 5 × 10^−8^ and *r*^2^<0.001, we selected 116 and 16 SNPs associated with LST and MVPA, respectively, for subsequent analysis (Tables S3E–F and Table S8 in the **Online Supplementary Document**). In the primary IVW analysis, there was no evidence to support a causal effect of genetics related to LST and MVPA on any thyroid disease and thyroid hormone phenotype (Figures S1–2 and Table S9 in the **Online Supplementary Document**).

The IVW (OR = 2.053; 95% CI = 1.060, 3.977, *P* = 0.033) and MR-PRESSO (OR = 2.053; 95% CI = 1.359, 3.102, *P* = 0.008) analyses indicated a nominally significant positive association of genetically determined increased MVPA with hypothyroidism, but the multiple testing threshold was not exceeded (Figure S1 and Table S9 in the **Online Supplementary Document**). Similarly, the IVW (β = 0.153; 95% CI = 0.001, 0.305, *P* = 0.049) and the weighted median (β = 0.224; 95% CI = 0.015, 0.433, *P* = 0.036) analyses indicated only a nominally significant positive association of genetically determined increased MVPA with TSH (Figure S2 and Table S7 in the **Online Supplementary Document**). None of the MR analyses were affected by directional pleiotropy, and the MR-Egger intercept and MR-PRESSO global tests were not significant (all *P* > 0.05). We also did not observe heterogeneity in our analyses (all *P* > 0.05). However, the funnel plot of MR analysis between MVPA and hypothyroidism and between MVPA and TSH showed asymmetry (Figures S3–8 in the **Online Supplementary Document**). The leave-one-out plot demonstrated that the causal link between MVPA and hypothyroidism was driven by several potentially influential SNPs (rs568546, rs13201721, rs1691471, rs7613360, and rs9420), while the causal link between MVPA and TSH was driven by several SNPs (rs7613360, rs12357890, rs385301, rs13201721, rs4352559, rs568546, rs1625595, and rs1691471); thus, we drew a cautious conclusion from these findings (Figures S3–8 in the **Online Supplementary Document**).

## DISCUSSION

This is the first MR analysis to examine the association between thyroid function and PA/SB. Using the GWAS data, we examined the bidirectional causal relationship between hypothyroidism, hyperthyroidism, TSH, and FT4, as well as self-reported LST and MVPA. The results confirmed our hypotheses: in the forward direction, the MR analysis indicated a positive causal relationship between genetically proxied TSH and LST, and this association persisted after adjusting for BMI in the MVMR analysis. Conversely, we could not establish a reverse causal relationship between thyroid function and PA/SB.

As a common lifestyle among modern individuals, SB is associated with numerous diseases [39]. Regular physical exercise in daily life has been shown to improve cardiovascular and metabolic health, emotional well-being, and cognitive function, and is considered one of the most effective lifestyle factors in reducing all-cause mortality rates [40–42]. Efforts to promote PA and reduce SB have received support from policymakers and the public [43,44]. Exploring the causal relationship between thyroid function and daily activity can provide new insights for addressing public health issues related to physical inactivity.

Previous studies on the relationship between thyroid function and PA have predominantly focussed on special populations such as athletes, military personnel, and older adults [16,45,46], with high exercise intensity and limited sample sizes. Studies investigating the relationship between thyroid function and daily PA in the general population are relatively scarce. Recently, a large-scale cross-sectional and cohort study reported no significant association between thyroid function and daily PA levels in the general population [12]. This finding is inconsistent with our previous understanding of TH and the existing conclusions from research on thyroid function and exercise. In the absence of randomised controlled trials, MR studies provide a higher level of efficacy for causal inference than observational studies. Therefore, we further examined the causal relationship between thyroid function and PA levels through univariable and multivariable MR analyses. We rigorously selected instrumental variable SNPs, excluded outliers, and minimised the influence of SNPs with reverse causal relationships on the overall MR analysis through MR Steiger filtering tests. We also explored the role of BMI in this relationship. Our findings support an increased risk of elevated LST with higher TSH levels within the normal range. One prior study reported no association between TSH, FT4, and PA among patients with hyperthyroidism defined [12]. Such variations in our conclusions might stem from differing ranges of TSH levels. Moreover, our study complements these findings by reporting no association between strictly defined patients with hyperthyroidism or hypothyroidism and PA levels. Furthermore, their study relied on data from the Rotterdam study, which included middle-aged (45 years) or older adults (≥55 years) [12], whereas our study extends these findings to a broader population.

A cross-sectional study using data from the NHANES also reported on the relationship between PA and thyroid function, showing a correlation between ln-transformed FT4, TSH, T4, and PA measured by the physical activity questionnaire (PAQ180) among participants after adjusting for BMI, age, and sex [14]. This is consistent with the results of our MVMR analysis after accounting for the influence of BMI. Further analyses, such as network MR using genetic instruments to study the mediating effects in the causal pathway, may provide new insights into the causal relationship. The PAQ180 asked participants to rate their overall daily activity levels on a scale of 1 to 4, with each consecutive number representing a higher activity level. However, in the PAD200 questionnaire, participants answered whether they engaged in vigorous PA with the categories ‘yes’, ‘no’, or ‘unable’, with the authors finding correlations between TSH, FT4, and engaging in vigorous PA. This represents a difference from the intensity of PA included in our study. Due to limitations in data resources, we focused solely on the relationship between thyroid function and MVPA as well as LST, which may be a limitation. Additionally, we only examined the effect of endogenous thyroid hormones on PA/SB, excluding patients undergoing thyroid medication treatment. Further investigation is needed to explore the impact of levothyroxine treatment on PA.

Cross-sectional studies cannot establish causality and cannot exclude reverse causality, while bidirectional MR analyses such as ours have certain advantages in interpreting the direction of causality. In the process of reverse direction MR analysis, we identified some SNPs with reverse causal relationships using the MR Steiger test of directionality. However, in the forward direction MR analysis, all SNPs were identified as ‘true’ in step two of MR Steiger filtering test results. After excluding SNPs with reverse causal relationships, we found no significant impact of LST and MVPA on thyroid function. Therefore, we speculate that the level of self-reported daily PA does not increase or decrease the risk of thyroid diseases. Although some intervention studies in animals and humans have found that PAs targeting resistance, endurance, and explosive training may influence thyroid hormone levels [47–49], this may be a temporary effect due to excessive exercise intensity [49].

Although MR analysis is considered an effective method for causal inference in genetic statistics, we must interpret and report our findings with caution due to certain limitations. First, false-positive results caused by sample overlap should be excluded or mitigated [50]. Second, although we conducted tests such as MR-Egger intercept [35] and stepwise screening with MR-PRESSO [34] to minimise the potential bias caused by pleiotropy, we cannot claim that all pleiotropic effects in the MR setting were eliminated. Finally, all participants included in our study were of European descent. While this reduces the heterogeneity in the MR analysis and increases the reliability of the results, it also limits the generalisability of the conclusions to other populations.

## CONCLUSIONS

This study is the first to report the relationship between thyroid function and PA/SB using univariable and multivariable MR. Genetically determined increases in TSH may be causally associated with higher levels of LST. The overall effect of TSH on LST does not appear to be mediated by BMI. We found no association between hyperthyroidism or hypothyroidism and PA/SB, but did not observe any impact of PA/SB on thyroid function and thyroid hormones in the reverse directions.

## Additional material


Online Supplementary Document
