# Supplementary material for: Thyroid function, physical activity and sedentary behaviour: A bidirectional two-sample Mendelian randomisation study
Source: J Glob Health. 2024 Sep 27;14:04154. doi: 10.7189/jogh.14.04154 (PMC11426939; doi:10.7189/jogh.14.04154)
Supplement: Online Supplementary Document [file jogh-14-04154-s001.zip › jogh-14-04154-s001.pdf]

Supplementary Figures

Exposures	Outcomes	Step	MR methods	No. of SNPs	OR (95%CI)	p
LST	Hypothyroidism	2	IVW (fixed effects)	82	1.130 (0.887-1.439)	0.321
		2	MR-Egger	82	1.088 (0.342-3.468)	0.886
		2	Weighted median	82	1.171 (0.833-1.647)	0.364
		2	MR-PRESSO	82	1.130 (0.910-1.403)	0.271
LST	Hyperthyroidism	2	IVW (fixed effects)	86	1.002 (0.942-1.065)	0.962
		2	MR-Egger	86	1.049 (0.775-1.421)	0.756
		2	Weighted median	86	0.999 (0.915-1.091)	0.990
		2	MR-PRESSO	86	1.002 (0.952-1.054)	0.954
MVPA	Hypothyroidism	2	IVW (fixed effects)	10	**2.053 (1.060-3.977)**	**0.033***
		2	MR-Egger	10	2.748 (0.126-59.73)	0.538
		2	Weighted median	10	2.084 (0.879-4.941)	0.095
		2	MR-PRESSO	10	**2.053 (1.359-3.102)**	**0.008†**
MVPA	Hyperthyroidism	2	IVW (fixed effects)	11	0.928 (0.423-2.035)	0.852
		2	MR-Egger	11	1.363 (0.027-69.695)	0.881
		2	Weighted median	11	1.086 (0.395-2.986)	0.873
		2	MR-PRESSO	11	0.928 (0.570-1.512)	0.771

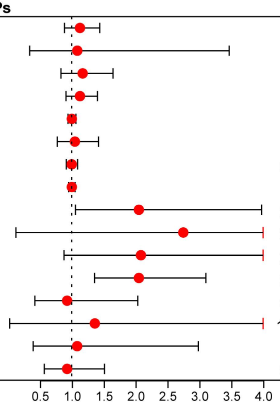

Fig. S1: MR analysis evaluating the causal effects of LST and MVPA on thyroid disease.

Step[#]: 2, MR analysis after removing the SNPs with FALSE in the MR Steiger test of directionality. LST = leisure screen time; IVW = Inverse variance weighted; MR = Mendelian randomization; MR-PRESSO = Mendelian randomization pleiotropy residual sum and outlier; MVPA = moderate-to-vigorous intensity physical activity during leisure time; SNPs = single nucleotide polymorphisms.

Bold,*$P < 0.05$. †$P < 0.01$. ‡$P < 0.001$.

Exposures	Outcomes	Step	MR methods	No. of SNPs	β (95%CI)	p
LST	TSH	2	IVW (fixed effects)	82	0.014 (-0.045-0.073)	0.630
		2	MR-Egger	82	0.081 (-0.197-0.360)	0.568
		2	Weighted median	82	0.040 (-0.046-0.126)	0.360
		2	MR-PRESSO	82	0.014 (-0.039-0.068)	0.594
LST	FT4	2	IVW (fixed effects)	85	-0.002 (-0.065-0.061)	0.954
		2	MR-Egger	85	-0.047 (-0.351-0.257)	0.762
		2	Weighted median	85	-0.003 (-0.091-0.085)	0.941
		2	MR-PRESSO	85	-0.002 (-0.054-0.05)	0.944
MVPA	TSH	2	IVW (fixed effects)	11	**0.153 (0.001-0.305)**	**0.049***
		2	MR-Egger	11	0.616 (-0.177-1.408)	0.162
		2	Weighted median	11	**0.224 (0.015-0.433)**	**0.036***
		2	MR-PRESSO	11	0.153 (-0.010-0.315)	0.095
MVPA	FT4	2	IVW (fixed effects)	11	-0.105 (-0.270-0.060)	0.210
		2	MR-Egger	11	-0.172 (-0.940-0.596)	0.672
		2	Weighted median	11	-0.102 (-0.312-0.108)	0.339
		2	MR-PRESSO	11	-0.105 (-0.219-0.008)	0.100

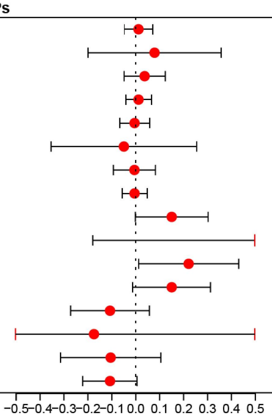

Fig. S2: MR analysis evaluating the causal effects of LST and MVPA on thyroid hormones.

Step#: 2, MR analysis after removing the SNPs with FALSE in the MR Steiger test of directionality.

FT4 = free thyroxine; LST = leisure screen time; IVW = Inverse variance weighted; MR = Mendelian randomization; MR-PRESSO = Mendelian randomization pleiotropy residual sum and outlier; MVPA = moderate-to-vigorous intensity physical activity during leisure time; SNPs = single nucleotide polymorphisms; TSH = thyrotropin.

Bold, **P* < 0.05. †*P* < 0.01. ‡*P* < 0.001.

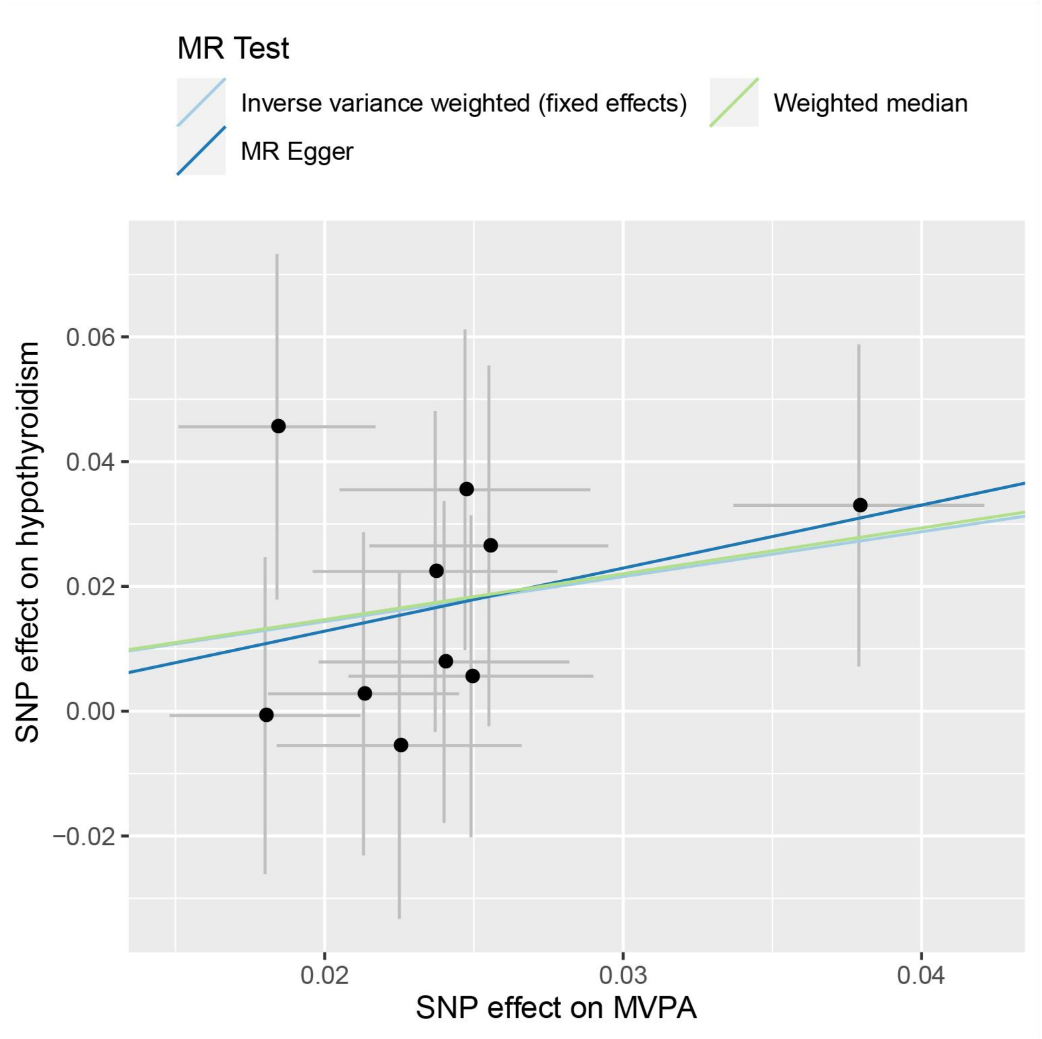

Figure S3: Scatter plots for MR estimates of the causal effect of MVPA on hypothyroidism.

MR, Mendelian randomization; MVPA, moderate-to-vigorous intensity physical activity during leisure time; SNP, single nucleotide polymorphism.

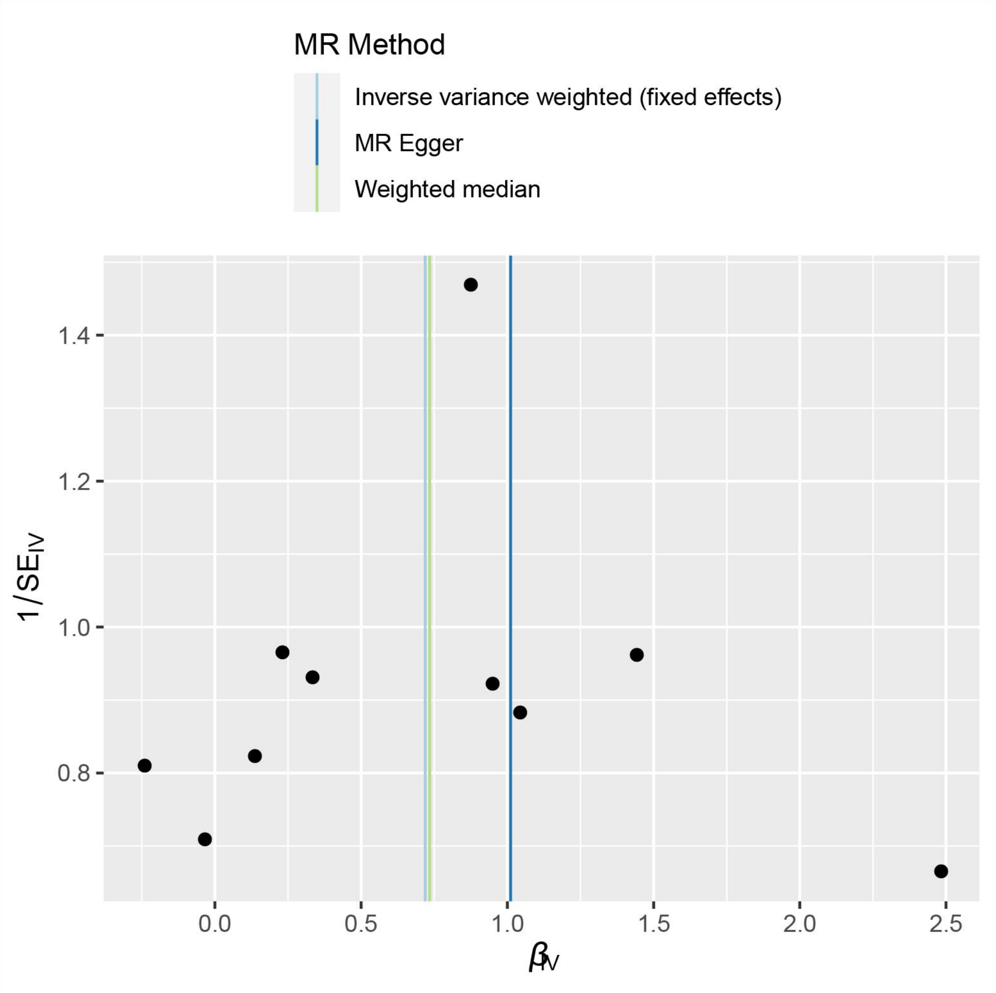

Figure S4: Funnel plots for MR estimates of the causal effect of MVPA on hypothyroidism.

MR, Mendelian randomization; MVPA, moderate-to-vigorous intensity physical activity during leisure time; SNP, single nucleotide polymorphism.

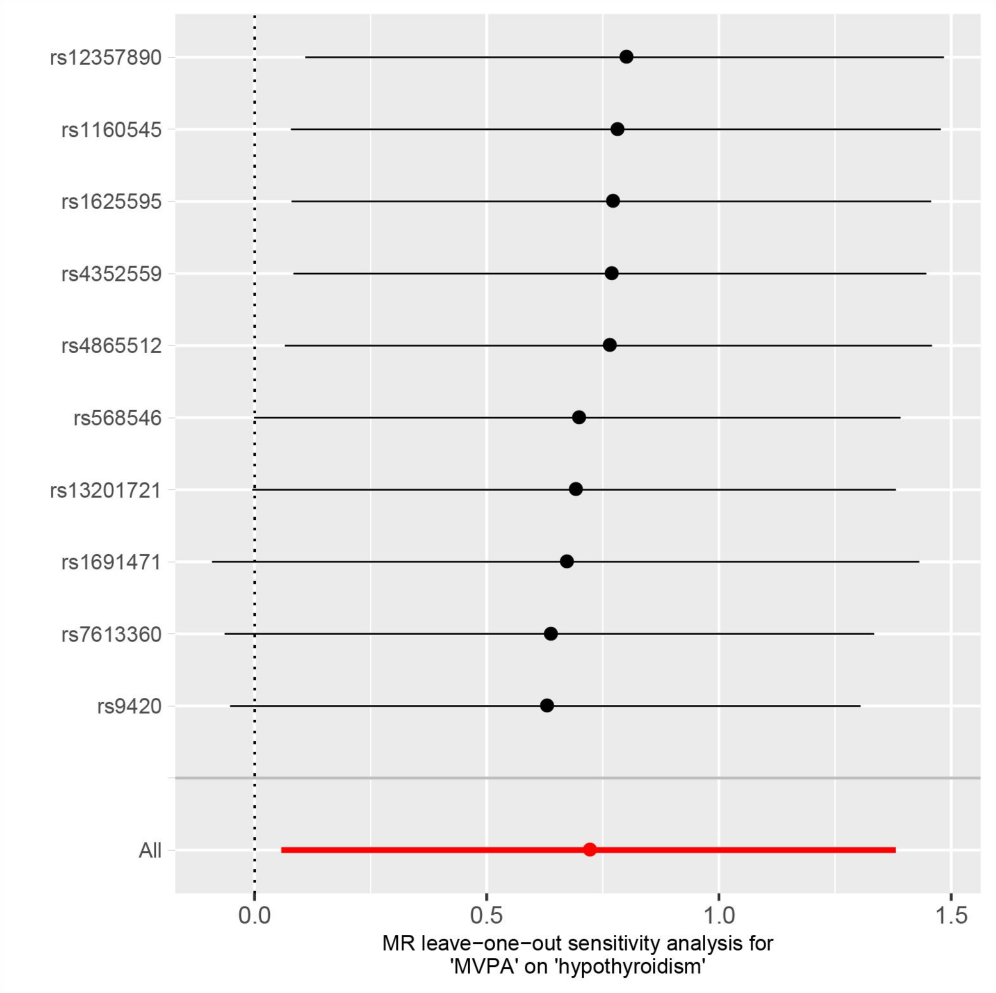

Figure S5: Plots of leave-one-out analysis for MR estimates of the causal effect of MVPA on hypothyroidism.

MR, Mendelian randomization; MVPA, moderate-to-vigorous intensity physical activity during leisure time; SNP, single nucleotide polymorphism.

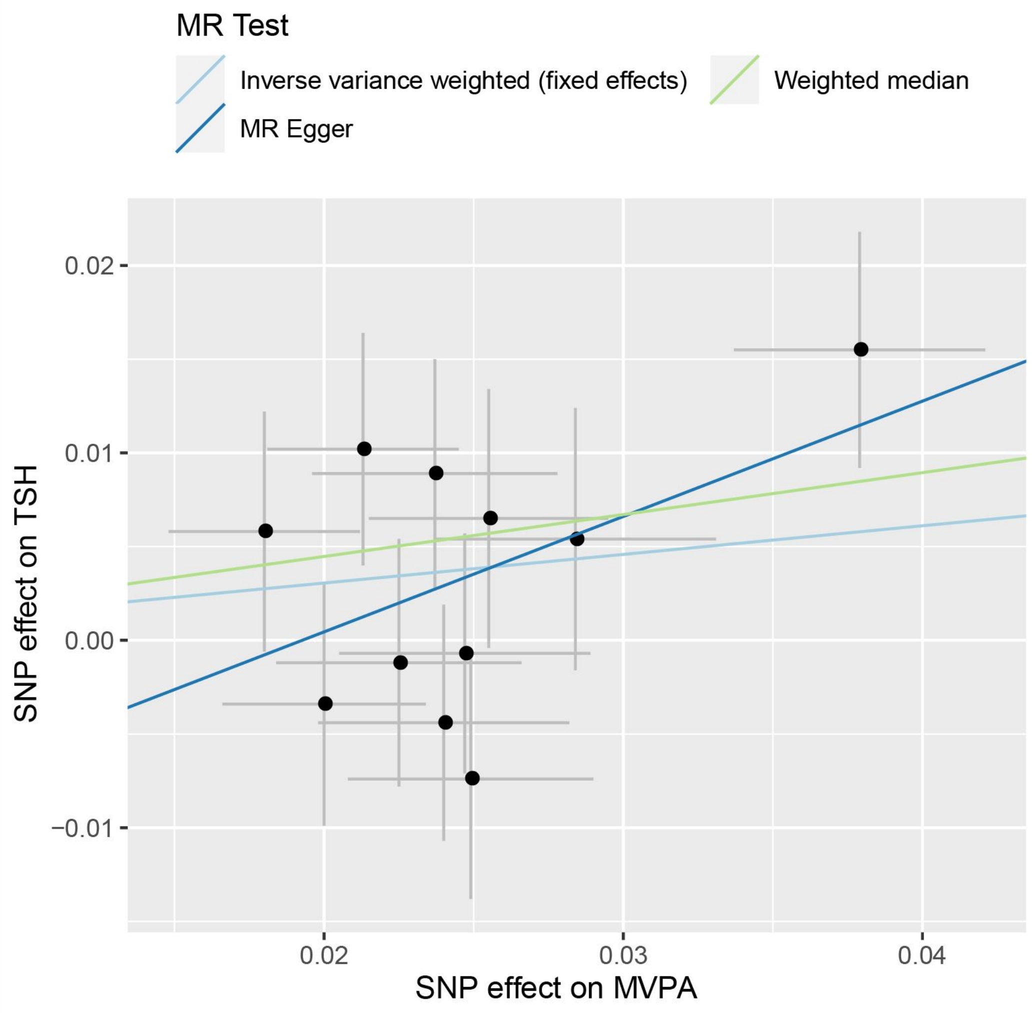

Figure S6: Scatter plots for MR estimates of the causal effect of MVPA on TSH.

MR, Mendelian randomization; MVPA, moderate-to-vigorous intensity physical activity during leisure time; SNP, single nucleotide polymorphism; TSH, thyrotropin.

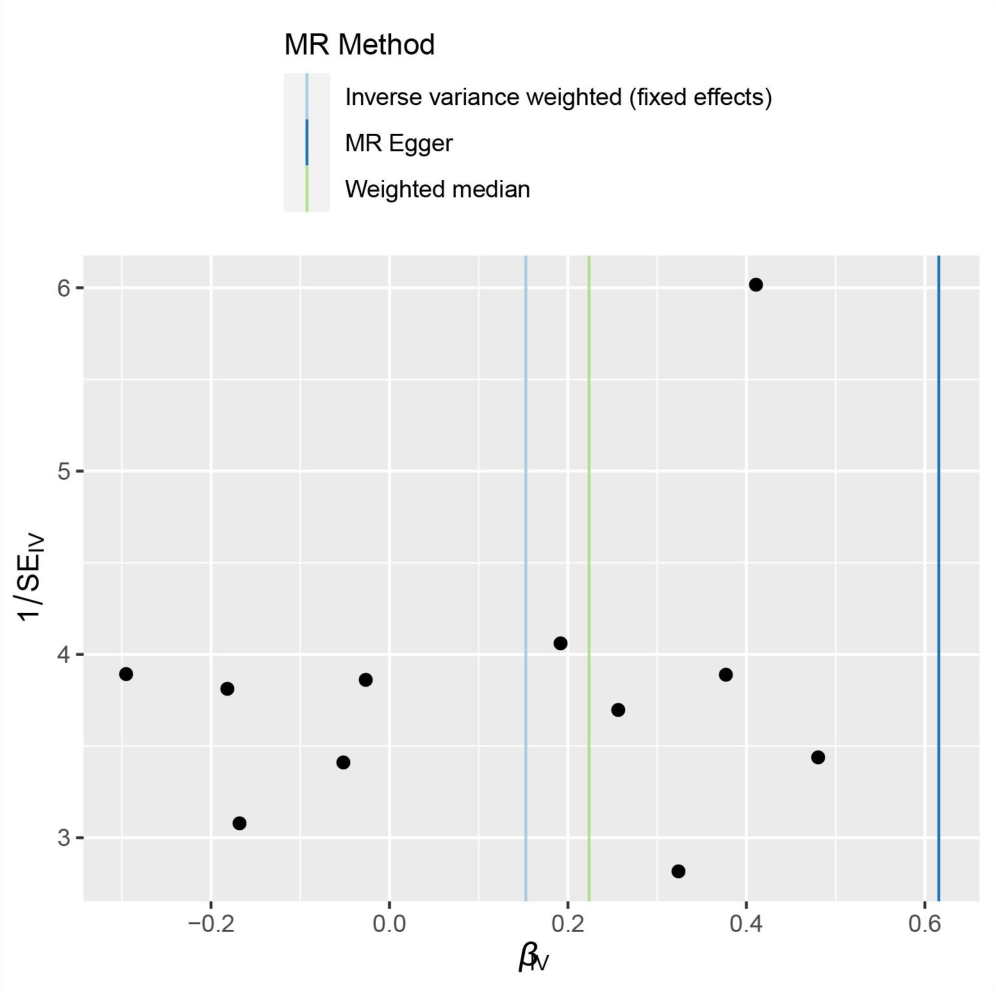

Figure S7: Funnel plots for MR estimates of the causal effect of MVPA on TSH.

MR, Mendelian randomization; MVPA, moderate-to-vigorous intensity physical activity during leisure time; SNP, single nucleotide polymorphism; TSH, thyrotropin.

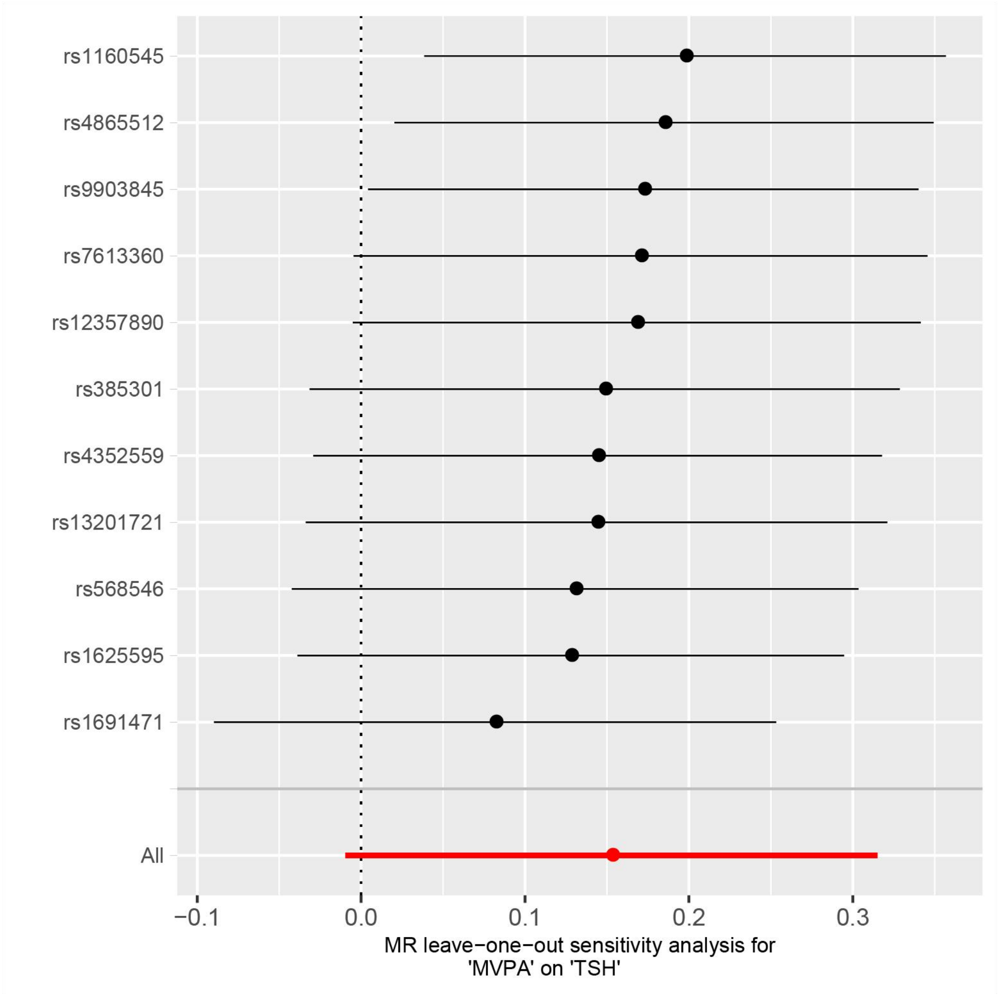

Figure S8: Plots of leave-one-out analysis for MR estimates of the causal effect of MVPA on TSH.

MR, Mendelian randomization; MVPA, moderate-to-vigorous intensity physical activity during leisure time; SNP, single nucleotide polymorphism; TSH, thyrotropin.

Supplementary TableS1. STROBE-MR checklist of recommended items to address in reports of Mendelian randomization studies[1] [2]

Item No.	Section	Checklist item	Note/ Page No.
1	**TITLE and ABSTRACT**	Indicate Mendelian randomization (MR) as the study's design in the title and/or the abstract if that is a main purpose of the study	'Mendelian randomization' in title and abstract
	**INTRODUCTION**		
2	**Background**	Explain the scientific background and rationale for the reported study. What is the exposure? Is a potential causal relationship between exposure and outcome plausible? Justify why MR is a helpful method to address the study question	Lines 62-91
3	**Objectives**	State specific objectives clearly, including pre-specified causal hypotheses (if any). State that MR is a method that, under specific assumptions, intends to estimate causal effects	Lines 92-99
	**METHODS**		
4	**Study design and data sources**	Present key elements of the study design early in the article. Consider including a table listing sources of data for all phases of the study. For each data source contributing to the analysis, describe the following:	Main text: lines 101-171 Table 1, Figure1-2<br>Supplementary Table S3
	a)	Setting: Describe the study design and the underlying population, if possible. Describe the setting, locations, and relevant dates, including periods of recruitment,	

		exposure, follow-up, and data collection, when available.	
	b)	Participants: Give the eligibility criteria, and the sources and methods of selection of participants. Report the sample size, and whether any power or sample size calculations were carried out prior to the main analysis	
	c)	Describe measurement, quality control and selection of genetic variants	
	d)	For each exposure, outcome, and other relevant variables, describe methods of assessment and diagnostic criteria for diseases	
	e)	Provide details of ethics committee approval and participant informed consent, if relevant	
5	**Assumptions**	Explicitly state the three core IV assumptions for the main analysis (relevance, independence and exclusion restriction) as well assumptions for any additional or sensitivity analysis	Lines 126-132
6	**Statistical methods: main analysis**	Describe statistical methods and statistics used	
	a)	Describe how quantitative variables were handled in the analyses (i.e., scale, units, model)	Lines 113-177
	b)	Describe how genetic variants were handled in the analyses and, if applicable, how their weights were selected	

		c) Describe the MR estimator (e.g. two-stage least squares, Wald ratio) and related statistics. Detail the included covariates and, in case of two-sample MR, whether the same covariate set was used for adjustment in the two samples	
		d) Explain how missing data were addressed	
		e) If applicable, indicate how multiple testing was addressed	
7	**Assessment of assumptions**	Describe any methods or prior knowledge used to assess the assumptions or justify their validity	Method Lines 134-171<br>Introduction
8	**Sensitivity analyses and additional analyses**	Describe any sensitivity analyses or additional analyses performed (e.g. comparison of effect estimates from different approaches, independent replication, bias analytic techniques, validation of instruments, simulations)	Lines 134 - 171
9	**Software and pre-registration**		
		a) Name statistical software and package(s), including version and settings used	Lines 173-177
		b) State whether the study protocol and details were pre-registered (as well as when and where)	
	**RESULTS**		

10	**Descriptive data**		
	a)	Report the numbers of individuals at each stage of included studies and reasons for exclusion. Consider use of a flow diagram	Lines 181-187<br>Figure 2
	b)	Report summary statistics for phenotypic exposure(s), outcome(s), and other relevant variables (e.g. means, SDs, proportions)	Supplementary Tables S3, S4, S6 and S8.
	c)	If the data sources include meta-analyses of previous studies, provide the assessments of heterogeneity across these studies	Not applicable, since in this study we used genome-wide summary statistics from previously published genome-wide association studies.
	d)	For two-sample MR:<br>i. Provide justification of the similarity of the genetic variant-exposure associations between the exposure and outcome samples<br>ii. Provide information on the number of individuals who overlap between the exposure and outcome studies	There is no sample overlap between the exposure and outcome studies
11	**Main results**		
	a)	Report the associations between genetic variant and exposure, and between genetic variant and outcome, preferably on an interpretable scale	Main text:<br>Lines: 188-236<br>Figure 3, Figure 5,
	b)	Report MR estimates of the relationship between exposure and outcome, and the measures of uncertainty from the MR analysis, on an interpretable scale, such as	

			odds ratio or relative risk per SD difference	Supplementary Tables S5, S7 and S9<br>Supplementary Figure 1 and Figure 2
		c)	If relevant, consider translating estimates of relative risk into absolute risk for a meaningful time period	
		d)	Consider plots to visualize results (e.g. forest plot, scatterplot of associations between genetic variants and outcome versus between genetic variants and exposure)	
12	**Assessment of assumptions**			
		a)	Report the assessment of the validity of the assumptions	Supplementary Tables S2
		b)	Report any additional statistics (e.g., assessments of heterogeneity across genetic variants, such as I^2, Q statistic or E-value)	Supplementary Tables S3, S5 and S7
13	**Sensitivity analyses and additional analyses**			
		a)	Report any sensitivity analyses to assess the robustnes<br>s of the main results to violations of the assumptions	Lines 188-236<br>Supplementary Tables S2
		b)	Report results from other sensitivity analyses or additional analyses	Figure 4

		c)	Report any assessment of direction of causal relationship (e.g., bidirectional MR)	Supplementary Tables S5, S7 and S9
		d)	When relevant, report and compare with estimates from non-MR analyses	
		e)	Consider additional plots to visualize results (e.g., leave-one-out analyses)	
	**DISCUSSION**			
14	**Key results**		Summarize key results with reference to study objectives	Lines 238-243
15	**Limitations**		Discuss limitations of the study, taking into account the validity of the IV assumptions, other sources of potential bias, and imprecision. Discuss both direction and magnitude of any potential bias and any efforts to address them	Lines 292-299
16	**Interpretation**			
		a)	Meaning: Give a cautious overall interpretation of results in the context of their limitations and in comparison with other studies	Lines 250-282<br>Lines 247-249
		b)	Mechanism: Discuss underlying biological mechanisms that could drive a potential causal relationship between the investigated exposure and the outcome, and whether the gene-environment equivalence assumption is reasonable. Use causal language carefully, clarifying that IV estimates may provide causal effects only under certain assumptions	
		c)	Clinical relevance: Discuss whether the results have clinical or public policy relevance, and to what extent they inform effect sizes of possible interventions	

17	**Generalizability**	Discuss the generalizability of the study results (a) to other populations, (b) across other exposure periods/timings, and (c) across other levels of exposure	Lines 250-282
	**OTHER INFORMATION**		
18	**Funding**	Describe sources of funding and the role of funders in the present study and, if applicable, sources of funding for the databases and original study or studies on which the present study is based	Acknowledgments<br>Funding
19	**Data and data sharing**	Provide the data used to perform all analyses or report where and how the data can be accessed, and reference these sources in the article. Provide the statistical code needed to reproduce the results in the article, or report whether the code is publicly accessible and if so, where	Main text: Table1<br>Lines 309-313<br>The code related to this article will not be made public.
20	**Conflicts of Interest**	All authors should declare all potential conflicts of interest	The authors declare that there is no conflict of interest.

This checklist is copyrighted by the Equator Network under the Creative Commons Attribution 3.0 Unported (CC BY 3.0) license.

1. Skrivankova VW, Richmond RC, Woolf BAR, Yarmolinsky J, Davies NM, Swanson SA, et al. Strengthening the Reporting of Observational Studies in Epidemiology using Mendelian Randomization (STROBE-MR) Statement. JAMA. 2021;under review.

2. Skrivankova VW, Richmond RC, Woolf BAR, Davies NM, Swanson SA, VanderWeele TJ, et al. Strengthening the Reporting of Observational Studies in Epidemiology using Mendelian Randomisation (STROBE-MR): Explanation and Elaboration. BMJ. 2021;375:n2233.

8

9

Table S2. Results of sensitivity analyses testing for heterogeneity by Cochrane's Q test and directional pleiotropy by MR-Egger intercept test and MR-PRESSO

Exposures	Outcomes	Step#	Cochran's Q Test		MR-Egger			MRPRESSO Global test	
			df	*p*	*Intercept*	*SE*	*p*	*RSSobs*	*p*
Hypothyroidism	LST	1	10	0.951	0	0.003	0.898	4.673	0.977
Hypothyroidism	MVPA	3	9	0.07	-0.002	0.005	0.769	19.055	0.074
Hyperthyroidism	LST	1	13	0.154	-0.003	0.004	0.502	4.673	0.973
Hyperthyroidism	MVPA	1	9	0.07	-0.002	0.005	0.769	24.22	0.141
TSH	LST	3	34	0.1	0	0.002	0.987	47.705	0.098
TSH	MVPA	4	32	0.458	0.001	0.002	0.772	38.151	0.343
FT4	LST	4	12	0.963	0.001	0.003	0.673	5.421	0.966
FT4	MVPA	4	16	0.149	0	0.003	0.971	23.719	0.196
LST	Hypothyroidism	2	81	0.905	0.001	0.016	0.948	66.448	0.903
LST	Hyperthyroidism	2	85	0.988	-0.001	0.004	0.758	59.824	0.987
LST	TSH	2	81	0.894	-0.002	0.004	0.631	67.196	0.895
LST	FT4	2	84	0.985	0.001	0.004	0.767	59.826	0.985
MVPA	Hypothyroidism	2	9	0.941	-0.007	0.039	0.854	4.101	0.945
MVPA	Hyperthyroidism	2	10	0.953	-0.01	0.05	0.85	4.621	0.959
MVPA	TSH	2	10	0.324	-0.012	0.01	0.273	14.624	0.321
MVPA	FT4	2	10	0.906	0.002	0.01	0.866	5.553	0.919

Abbreviation: df = degree of freedom; LST = leisure screen time; MR-PRESSO = Mendelian randomization pleiotropy residual sum and outlier; MVPA = moderate-to-vigorous intensity physical activity during leisure time; RSSobs = observed residual sum of squares; SE = standard error; SNPs = single nucleotide polymorphisms; TSH = thyrotropin; MR = Mendelian randomization; $p < 0.05$ indicates significant effects.

Step#: 1, MR analysis with the complete selected SNPs; 2, MR analysis after removing the SNPs with FALSE in the MR Steiger test of

directionality; 3, MR analysis after removing the SNPs with *p* value less than threshold (0.05) in the MR-PRESSO outlier test; 4, MR analysis after removing all the SNPs with *p* value less than 1 in the MR-PRESSO outlier test.
